# Tea, coffee, caffeine intake and the risk of cardio-metabolic outcomes: findings from a population with low coffee and high tea consumption

**DOI:** 10.1186/s12986-019-0355-6

**Published:** 2019-05-03

**Authors:** Zahra Gaeini, Zahra Bahadoran, Parvin Mirmiran, Fereidoun Azizi

**Affiliations:** 1 Nutrition and Endocrine Research Center, Research Institute for Endocrine Sciences, Shahid Beheshti University of Medical Sciences, P.O. Box 19395-4763, No. 24, Shahid-Erabi St., Yeman St., Velenjak, Tehran, Iran; 2 Department of Clinical Nutrition and Dietetics, Faculty of Nutrition Sciences and Food Technology, National Nutrition and Food Technology Research Institute, Shahid Beheshti University of Medical Sciences, P.O. Box 1981619573, No.47, Shahid Hafezi St. Farahzadi Blvd. Sharak-e- Ghods., Tehran, Iran; 3 Endocrine Research Center, Research Institute for Endocrine Sciences, Shahid Beheshti University of Medical Sciences, Tehran, Iran

**Keywords:** Tea, Coffee, Caffeine, Cardiovascular disease, Chronic kidney disease, Hypertension

## Abstract

**Background:**

This study aimed to assess the potential effects of long-term intake of caffeine and habitual consumption of coffee and tea on the occurrence of cardio-renal events among an Iranian population with low coffee and high tea consumption.

**Methods:**

Adult participants of the Tehran Lipid and Glucose Study (2006–2008 to 2012–2014) who met the study inclusion criteria, were recruited. Habitual dietary intakes were assessed using a validated food frequency questionnaire. Demographics, anthropometrics, blood pressure, and biochemical variables were evaluated at baseline and during follow-up examinations. Multivariate Cox proportional hazard and logistic regression models adjusted for potential confounders were used to estimate the risk of cardiovascular disease (CVD), hypertension (HTN) and chronic kidney disease (CKD).

**Results:**

During median 6 years of follow-up, the incidence rate of CVD outcomes, HTN, and CKD were 3.3%, 15.5%, and 17.9%, respectively. The risk of CVD was increased more than two-fold in the highest tertile of tea consumption (HR = 2.44, 95% confidence interval, CI = 1.40–4.27; P for trend = 0.001), and caffeine intakes (HR = 2.22, 95% CI = 1.23–4.01; P for trend = 0.005). A 42% lower incidence of CVD was observed in coffee drinkers, compared to non-drinkers (HR = 0.58, 95% CI = 0.36–0.93; P for trend = 0.023). No significant association was observed between tea, coffee or caffeine intakes and the risk of HTN or CKD.

**Conclusions:**

Findings of our study support previous data regarding the protective effects of coffee on CVD. Contrary to the previous studies, we found that higher intakes of tea and caffeine, mainly originated from tea in our population, may increase risk of CVD events. It may be related to the type of tea and its preparation methods, additives or artificial colors in tea consumed in Iran, and sweets or sugar that mostly consumed accompanied by tea. Also, genetic variants of the liver enzymes may modify the association of dietary caffeine sources and incidence of CVD. Further prospective studies with incorporation of different population with different dietary habits and genetic backgrounds are needed to clarify the contradictions.

## Background

Cardiovascular disease (CVD), a major cause of early death, is a global public health concern [1]. CVDs, defined as a group of disorders of the heart and blood vessels, include coronary heart disease (CHD), cerebrovascular disease, peripheral arterial disease, rheumatic heart disease, congenital heart disease, deep vein thrombosis and pulmonary embolism [1]. High blood pressure (HTN) is an important risk factor for CVD, which increases all-cause mortality in older adults [2]. Chronic kidney disease (CKD) and CVD are closely interrelated; subjects with renal dysfunction show higher risk of CVD-related mortality [3–5]. Chronic kidney disease is associated with CKD, independent of traditional CVD risk factors such as HTN, hyperlipidemia, and diabetes [3].

Caffeine, the most studied pharmacologically active substance in coffee and tea, is well-known for its beneficial and/or adverse effects on human health [6, 7]. A moderate daily dose of caffeine have been shown to be associated with decreased risk of metabolic disorders [7, 8]. Some epidemiologic studies suggested that moderate habitual intakes of caffeine, coffee and tea may have protective effects against development of type 2 diabetes [9, 10], dementia and Alzheimer disease [11], metabolic syndrome [12] and non-alcoholic fatty liver disease [13]. We previously observed that the risk of pre-diabetes and type 2 diabetes was lower in coffee drinkers compared to non-drinkers [14]. Several observational studies have also reported the beneficial effects of tea, coffee and/or caffeine intake on CVD [15–17]. A meta-analysis of prospective cohorts, assessing the long term effects of coffee on CVD, reported a significant inverse association in moderate but not heavy coffee consumption [18]. Similarly, moderate coffee consumption is known to decrease risk of stroke [19]. Relation between coffee, caffeine and risk of HTN have demonstrated conflicting results [20–22]. Long-term effects of caffeine intake on renal function have also been less documented. In a prospective cohort study, coffee intakes more than 1 cup per day were associated with decreased risk of the development of chronic kidney disease [23].

In this study, we aimed to assess the potential long-term effects of dietary caffeine, as well as coffee or tea consumption on the risk of CVD, HTN and CKD, among Iranian adults with low-coffee and high-tea consumption. This population-based cohort study was conducted within the framework of the Tehran Lipid and Glucose Study (TLGS).

## Methods

### Study population

**Fig. 1:**
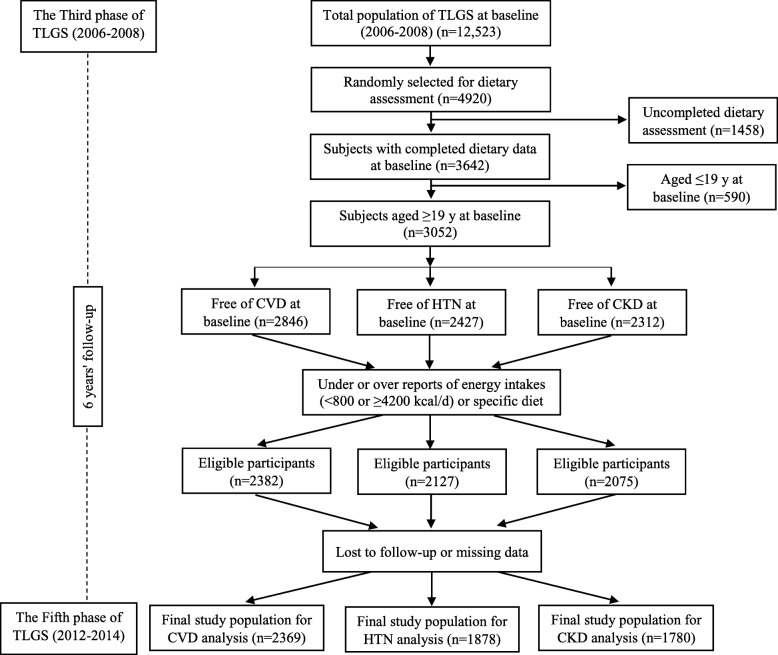
The diagram of the study

The present study was conducted using data collected from the TLGS, an ongoing community-based prospective study conducted on a sample of residents from district 13, Tehran, Iran [24]. The first phase of the TLGS was initiated in 1998 with participation of 15,005 individuals. Collecting data from participants had been repeated every 3 years [25]. We recruited 3687 men and women who had participated in the third TLGS phase (2006–2008), with complete dietary data (completed FFQ). The characteristics of participants who completed the FFQ were similar to those of the total population in the third phase of TLGS [26]. For the current analysis, 3052 adult men and women (age ≥ 19 years) with complete baseline data (demographics, anthropometrics, biochemical and dietary data), were included. After exclusion of participants with CVD, HTN or CKD outcomes, as well as exclusion of participants with under- or over-reports of energy intakes, or with specific diets, and participants who lost to follow-up or have missing data, final study population for CVD, HTN and CKD were 2369, 1878 and 1780 adults, respectively (Fig. 1); the remaining eligible participants were followed up to the fifth phase of TLGS (2012–2014). Mean period of follow-up for CVD outcomes, CKD and HTN was 6.7, 6.4 and 5.8 years from the baseline examination, respectively.

### Anthropometric and demographic measures

Anthropometric data were collected by the trained interviewers. Body weight was measured, to the nearest 100 g, using digital scales (Seca, Hamburg, Germany), while the subjects were minimally clothed and without shoes. Height was recorded to the nearest of 0.5 cm, in a standing position, without shoes, using a tape meter. Body mass index (BMI) calculated as weight (kg) divided by height in square (m^2^). Waist circumference was measured using a soft measuring tape, midway over light clothing, between the lower border of the ribs and the iliac crest at the widest portion, without any pressure to the body.

For measurements of systolic (SBP) and diastolic (DBP) blood pressures, after a 15-min rest in a sitting position, two measurements of blood pressure were taken on the right arm, using a standard mercury sphygmomanometer calibrated by the Iranian Institute of Standards and Industrial Researches [27]. Depending on the participants arm circumference, a regular adult or large cuff was used. The cuff was placed at heart level on the participant’s right arm and inflated at an increment rate, until the cuff pressure was 30 mmHg above the level at which the radial pulse disappeared. There was at least a 30-s interval between the two blood pressure measurements and mean of the two measurements was considered as the participant’s blood pressure; participants were asked to avoid tea or coffee consumption, physical activity, and smoking and were also asked to empty their bladder 30 min prior to the measurements. Physical activity was assessed using the Modifiable Activity Questionnaire (MAQ); the frequency and time spent on light, moderate, hard and very hard intensity activities according to the list of common activities of daily life over the past year were documented. Reliability and convergent validity of the Persian version of the MAQ has previously been investigated. Physical activity levels were expressed as metabolic equivalent hours per week (MET-h/wk) [28].

### Biochemical measures

Blood samples were taken after 12 to 14 h of overnight fasting, between 7:00 and 9:00 AM. Fasting plasma glucose (FPG) and 2-h post-challenge plasma glucose (2 h-PCPG) were assayed using glucose oxidase. Measurement of serum triglycerides was done by enzymatic colorimetric method using glycerol phosphate oxidase. High-density lipoprotein-cholesterol (HDL-C) was measured after precipitation of the Apo-lipoprotein B containing lipoproteins with phosphotungstic acid. All blood analysis was done at the research laboratory of the TLGS, using Pars Azmoon kits (Pars Azmoon Inc., Tehran, Iran) and a Selectra 2 auto-analyzer (Vital Scientific, Spankeren, The Netherlands). Serum creatinine levels were measured by kinetic colorimetric Jaffe methods. Both inter- and intra-assay coefficients of variations (CVs) were less than 5%.

### Dietary assessment

A validated 168-item food frequency questionnaire (FFQ) was used to assess typical food intakes over the previous year. Trained dietitians, with at least 5 years of experience in the TLGS survey, asked participants to designate their intake frequency for each food item consumed during the past year on a daily, weekly, or monthly basis. Portion sizes of consumed foods reported in household measures were then converted to grams [26]. Participants were questioned about frequency of drinking coffee or tea in the preceding year, considering a given portion size (cups per day or week or month). Caffeine intake was calculated as mg/day, from the sum of caffeine content in tea, coffee, soft drinks and chocolates. We did not collect any information on the type of coffee or tea and their preparation methods in the current study.

The validity of the food frequency questionnaire has been previously evaluated by comparing food groups and nutrient values determined from the questionnaire with values estimated from the average of twelve 24-h dietary recall surveys and the reliability has been assessed by comparing energy and nutrient intakes from two FFQs; Pearson correlation coefficients and intra-class correlation for energy and nutrients showed that the correlation coefficients between the FFQ and multiple 24 h recalls were 0.53 and 0.39, and those between the two FFQs were 0.59 and 0.60 in males and females, respectively. So it was shown acceptable agreements between the FFQs and twelve 24-h dietary recall surveys, and FFQ1 and FFQ2. The correlation coefficients between the FFQ and multiple 24 h recalls were reported 0.39 and 0.47 for carbohydrate, 0.65 and 0.50 for protein, 0.59 and 0.38 for total fat in males and females, respectively. Those between the two FFQs were 0.45 and 0.47 for carbohydrate, 0.79 and 0.69 for protein, 0.43 and 0.42 for total fat in males and females, respectively [29].

### Definition of terms and outcomes

Hypertension was defined as SBP ≥ 140 mmHg or DBP ≥ 90 mmHg, or self-reported usage of blood pressure lowering medications [30]. Details of the data collection for CVD outcomes have been described elsewhere [31]. Cardiovascular disease outcomes were defined as any CHD event, stroke (a new neurological deficit that lasted ≥24 h), or CVD deaths (definite fatal myocardial infraction (MI), definite fatal CHD, and definite fatal stroke) [32]. CHD events included cases of definite MI (diagnostic ECG and biomarkers), probable MI (positive ECG findings plus cardiac symptoms or signs plus missing biomarkers or positive ECG findings plus equivocal biomarkers), and angiographic proven CHD. History of CVD was defined as previous ischemic heart disease and/or cerebro-vascular accidents.

Chronic kidney disease was defined as estimated GFR (eGFR) < 60 mL/min per 1.73 m^2^ [33]. To calculate eGFR, the CKD-EPI creatinine equation, developed by the Chronic Kidney Disease Epidemiology Collaboration, was used. As a single equation CKD-EPI has been expressed as follows:

eGFR = 141 × min (S_cr_/κ,1)^α^ × max (S_cr_/κ, 1)^-1.209^ × 0.993^age^ × 1.018 [if female] × 1.159 [if black].

In this equation, S_cr_ is serum Cr in mg/dL; κ is 0.7 and 0.9 for men and women, respectively, α is − 0.329 and − 0.411 for men and women, respectively; min indicates the minimum of S_cr_/κ or 1, and max indicates maximum of S_cr_/κ or 1 [34].

### Statistical analyses

Mean and standard deviation (SD) values, and frequency (%) of baseline characteristics of participants were compared according to incidence of CVD, CKD and HTN, using analysis of variance or chi square test, respectively.

The incidence of CVD and HTN over the follow-up period was considered as a dichotomous variable (yes/no) in the models. Dietary intakes of caffeine, coffee and tea intake were entered in the models as both continuous and categorical variables. In the categorical model, intakes of caffeine and tea were categorized into tertiles, and the first tertile was considered as reference. Participants were categorized to two groups according to coffee drinking status (as “drinkers” and “non-drinkers”). In the continuous model, hazard ratio was calculated for each 100 mg/day caffeine, 1 cup/day tea and 1 cup/week coffee increases for each related variables.

Cox proportional hazards regression models with person-years as the underlying time metric were used to estimate HRs and 95% confidence intervals (CIs) for associations between intakes of caffeine, coffee and tea and the incidence of CVD. Time to event for CVD was defined as time to end of follow-up (censored cases) or time to having an event, whichever occurred first. We censored participants at the time of death due to non-CVD causes, at time of leaving the district, or end of study follow-up (March 2014). For the censored and lost to follow-up subjects, the survival time was the interval between the first and the last observation dates. The proportional hazard assumption of the multivariable Cox model was assessed using Schoenfelds global test of residuals. To estimate incidence of CKD and HTN multivariable logistic regression models were used.

To obtain the final multivariable models and determine confounding variables, we performed a univariate analysis. Variables with P_E_ less than 0.2 in the univariate analyses were selected as confounders. Potential confounders, adjusted in the Cox models, include CVD risk score (continuous) [35], total energy (kcal/d), fat and fiber intakes (g/d) for CVD outcomes. The CVD risk score calculated based on age, total cholesterol, HDL-C, SBP, treatment for HTN, smoking, and type 2 diabetes status, which has been validated among Iranian population [36]. Adjustment of CVD risk score, as a continuous potential risk factor of CVD events, improved the stability of our models due to the limited number of events during the study follow-up. For HTN and CKD [37], the models were adjusted for age (years), sex (male/female), BMI (kg/m^2^), triglyceride to HDL-C ratio, smoking (yes/no), total energy (kcal/d) and fat (g/d), and total fiber intake (g/d). Analyses of coffee were adjusted for tea, and vice versa. To assess the overall trends across increasing tertiles of caffeine or tea intake and each of three outcomes and to determine *P* values for trend, the median of each tertile of caffeine or tea was used as a continues variable in the regression models. CVD, HTN or CKD incidence was used as a dependent variable in the linear regression models. *P* values obtained from regression models were considered as *P* values for trend.

All statistical analyses were performed using the Statistical Package for Social Science (version 20; IBM Corp., Armonk, NY, USA) and STATA version 12 SE (Stata Corp LP, College Station, TX, USA), *P*-values < 0.05 being considered significant.

## Results

During an average of 6 years of follow-up, the incidence rate of CVD events, HTN, and CKD, were 3.3%, 15.5%, and 17.9%, respectively. In our study population, the main source of caffeine intakes was habitual drinking of tea (≈90%); other sources of dietary caffeine were coffee (4%), caffeinated soft drinks (4%) and chocolates (1%). Also, caffeine content of tea and coffee per 1 cup was estimated 50 and 65 mg, respectively.

Baseline characteristics of the participants are shown in Table 1. Compared with participants who had no CVD-related events, participants with CVD outcomes, tended to be older, more likely to be male and current smokers, had higher BMI, waist circumference, serum creatinine, creatinine clearance, eGFR, SBP, DBP, FPG and TG/HDL ratio, as well as higher daily intakes of tea and caffeine, at baseline (*P* for all < 0.05). Participants with HTN outcomes had higher age, BMI, waist circumference, SBP, DBP, FPG, TG/HDL ratio, serum creatinine, eGFR and higher intakes of tea and caffeine, in comparison to participants who did not have HTN at baseline (*P* for all < 0.05). Participants with CKD outcomes were more likely to be female and less smoker, had significantly higher BMI, waist circumference, SBP, DBP, FPG, TG/HDL ratio, and significantly lower values for eGFR. There was no significant difference in dietary intakes of tea, coffee and caffeine between the two groups of CKD outcome. Also, subjects in the highest tertile of tea consumption were significantly older, more likely to be male, had higher BMI, SBP and DBP, and they were more smoker, compared to subjects in the lowest tertile of tea consumption. In contrast, coffee drinkers were significantly younger, more likely to be female, had lower BMI, SBP and DBP, and they were more non-smoker, compared to non-drinkers.

**Table 1:** Baseline characteristics of the participants across the two groups with or without outcomes: Tehran Lipid and Glucose Study (2006–2008)

Variables	CVD		HTN		CKD	
	YES (*n* = 79)	NO (*n* = 2290)	YES (*n* = 291)	NO (*n* = 1587)	YES (*n* = 318)	NO (*n* = 1462)
Age (year)	58.5 ± 9.8	37.4 ± 12.8*	44.3 ± 12.2	35.1 ± 11.9^*^	34.3 ± 15.7	33.9 ± 15.3
Male (%)	68.4	42.6*	50.5	41.5*	35.2	42.1*
Smoking (%)	20.2	11.7*	2.1	3.0	5.8	9.5*
Physical activity (MET-h/week)	31.7 ± 46.0	36.1 ± 58.6	33.0 ± 52.9	35.2 ± 57.6	47.4 ± 68.9	43.9 ± 66.1
BMI (m^2^/kg)	28.4 ± 4.4	26.5 ± 4.8*	29.0 ± 4.6	25.8 ± 4.5*	28.5 ± 4.5	27.1 ± 4.8*
Waist circumference (cm)	97.4 ± 9.9	87.9 ± 13.3*	95.2 ± 10.6	85.8 ± 12.8*	95.0 ± 11.0	89.9 ± 13.3*
SBP (mmHg)	128 ± 19.0	109 ± 14.8*	115 ± 10.7	105 ± 11.2*	122 ± 19.9	112 ± 16.3*
DBP (mmHg)	79.9 ± 11.2	72.4 ± 10.3*	77.0 ± 7.4	69.7 ± 8.6*	74.7 ± 10.6	72.1 ± 10.4*
FPG (mg/dl)	104 ± 37.5	88.3 ± 16.1*	93.3 ± 23.0	86.7 ± 12.7*	106 ± 36.9	93.0 ± 23.7*
TG/HDL-ratio	5.1 ± 3.2	3.4 ± 2.7*	4.3 ± 2.9	3.2 ± 2.5*	4.4 ± 2.9	3.9 ± 3.2*
Serum creatinine (μmol/l)	102 ± 24.1	91.8 ± 13.1*	93.9 ± 16.2	91.4 ± 12.9*	91.9 ± 17.3	92.2 ± 15.9
Creatinine clearance (ml/min)	73.8 ± 19.5	90.7 ± 22.9*	91.0 ± 23.1	90.1 ± 21.8	94.3 ± 22.3	96.3 ± 25.4
eGFR (ml/min per 1.73m^2^)	67.5 ± 13.7	80.3 ± 13.5*	76.5 ± 12.9	81.6 ± 13.2*	68.9 ± 7.9	80.7 ± 12.5*
Tea intake (ml/d)	784 ± 601	605 ± 579*	690 ± 649	593 ± 587*	585 ± 595	567 ± 544
Coffee intake (ml/d)	12.5 ± 51.5	14.2 ± 62.6	9.9 ± 33.0	15.6 ± 72.0	12.0 ± 31.7	14.6 ± 59.7
Caffeine intake (mg/d)	162 ± 121	129 ± 115*	144 ± 129	126 ± 115*	125 ± 122	122 ± 110

Data are mean ± SE

*P < 0.05

*eGFR* estimated glomerular filtration rate, *FPG* fasting plasma glucose, *TG* triglyceride, *HDL* high-density lipoprotein, *MET* metabolic equivalent

The hazard ratios (with 95% CI) of CVD, HTN and CKD across categories of caffeine intake are shown in Table 2. After adjustment for confounding variables, a higher habitual intake of caffeine was associated with an over two-fold risk of CVD outcomes (HR = 2.22, 95% CI = 1.23–4.01; *P* for trend = 0.005). Further analysis indicated a 14% increased risk of CVD associated with each 100 mg/d increase in caffeine intake (HR = 1.14, 95% CI = 1.01–1.28). However, there were no significant associations between caffeine intake and HTN or CKD outcomes in both crude and adjusted models.

**Table 2:** The risk of cardio-renal outcomes across tertiles of caffeine intakes: Tehran Lipid and Glucose Study

Caffeine	Tertile 1	Tertile 2	Tertile 3	*P* for trend	Each 100 mg/d
CVD^a^	(< 60.25 mg/day)	(60.25–151.4 mg/day)	(> 151.4 mg/day)		
Crude	1.00	1.16 (0.63–2.14)	2.02 (1.16–3.50)*	0.007	1.16 (1.03–1.29)
Model 1	1.00	1.30 (0.69–2.45)	2.26 (1.27–4.02)*	0.003	1.16 (1.03–1.29)
Model 2	1.00	1.35 (0.71–2.56)	2.22 (1.23–4.01)*	0.005	1.14 (1.01–1.28)*
HTN ^b^	(< 60.60 mg/day)	(60.61–151.3 mg/day)	(> 151.3 mg/day)		
Crude	1.00	1.02 (0.75–1.40)	1.21 (0.89–1.64)	0.17	1.11 (1.02–1.22)*
Model 1	1.00	1.02 (0.73–1.43)	0.98 (0.70–1.36)	0.95	1.03 (0.93–1.14)
Model 2	1.00	1.02 (0.73–1.44)	0.98 (0.70–1.38)	0.94	1.03 (0.93–1.14)
CKD^c^	(< 56.16 mg/day)	(56.17–150.2 mg/day)	(> 150.2 mg/day)		
Crude	1.00	0.83 (0.62–1.12)	0.87 (0.65–1.17)	0.47	1.03 (0.93–1.14)
Model 1	1.00	0.81 (0.59–1.12)	0.87 (0.63–1.19)	0.49	1.03 (0.92–1.15)
Model 2	1.00	0.83 (0.60–1.14)	0.87 (0.63–1.21)	0.52	1.04 (0.93–1.16)

Data are hazard ratio (95% CI); proportional hazard Cox regression and logistic regression were used. *CI* confidence interval, *CKD* chronic kidney disease, *CVD* cardiovascular disease, *HTN* hypertension

Median of caffeine intake in the first, second and third tertile in CVD population was 78.79, 103.5 and 137.3 mg/day, respectively

Median of caffeine intake in the first, second and third tertile in HTN population was 51.20, 103.3 and 202 mg/day, respectively

Median of caffeine intake in the first, second and third tertile in CKD population was 50.93, 101.2 and 200.5 mg/day, respectively

^a^Model 1 was adjusted for CVD risk score; model 2 was additionally adjusted for dietary fat (g/d), fiber (g/d) and total energy (kcal/d)

^b^Model 1 was adjusted for sex, age, BMI, TGs to HDL-C ratio; model 2 was additionally adjusted for total energy intake (kcal/d)

^c^Model 1 was adjusted for sex, age, BMI, TGs to HDL-C ratio, and smoking; model 2 was additionally adjusted for dietary fat (g/d), fiber (g/d) and total energy (kcal/d)

*P <  0.05

The risk of CVD, HTN and CKD outcomes across tertile categories of tea intakes are shown in Table 3. In the crude model, a higher intake of tea was associated with increased risk of CVD and HTN (*P* for both < 0.05). In the fully adjusted model, an increased risk of CVD (HR = 2.44, 95% CI = 1.40–4.27, *P* for trend = 0.001) was observed in participants with highest intakes of tea and a 4% increased risk of CVD was observed corresponding to each one cup of tea per day (HR = 1.04, 95% CI = 1.00–1.07). There was no significant association between tea intakes and HTN or CKD outcomes in the adjusted models.

**Table 3:** The risk of cardio-renal outcomes across tertiles of tea intakes: Tehran Lipid and Glucose Study

Tea	Tertile 1	Tertile 2	Tertile 3	*P* for trend	Each cup/d
CVD^a^	(< 250 ml/day)	(250–750 ml/day)	(> 750 ml/day)		
Crude	1.00	1.19 (0.60–2.35)	2.37 (1.40–4.01)*	0.001	1.04 (1.01–1.07)
Model 1	1.00	1.28 (0.64–2.57)	2.52 (1.45–4.36)*	0.001	1.04 (1.01–1.07)
Model 2	1.00	1.30 (0.64–2.61)	2.45 (1.40–4.29)*	0.001	1.04 (1.00–1.07)*
HTN^b^	(< 250 ml/day)	(250–750 ml/day)	(> 750 ml/day)		
Crude	1.00	0.80 (0.56–1.15)	1.44 (1.09–1.91)*	0.003	1.03 (1.01–1.06)*
Model 1	1.00	0.83 (0.56–1.21)	1.09 (0.81–1.48)	0.39	1.01 (0.98–1.04)
Model 2	1.00	0.82 (0.56–1.21)	1.09 (0.80–1.49)	0.38	1.01 (0.98–1.04)
CKD^c^	(< 250 ml/day)	(250–750 ml/day)	(> 750 ml/day)		
Crude	1.00	0.93 (0.67–1.28)	0.97 (0.74–1.28)	0.87	1.01 (0.98–1.04)
Model 1	1.00	0.89 (0.64–1.25)	0.92 (0.69–1.23)	0.74	1.01 (0.98–1.04)
Model 2	1.00	0.89 (0.63–1.24)	0.92 (0.68–1.25)	0.78	1.01 (0.98–1.04)

Data are hazard ratio (95% CI); proportional hazard Cox regression and logistic regression were used. *CI* confidence interval, *CKD* chronic kidney disease, *CVD* cardiovascular disease, *HTN* hypertension

Median of tea intake in the first, second and third tertile was 250, 500 and 1000 ml/day, respectively

^a^Model 1 was adjusted for CVD risk score; model 2 was additionally adjusted for coffee (ml/day), dietary fat (g/d), fiber (g/d) and total energy (kcal/d)

^b^Model 1 was adjusted for sex, age, BMI, TGs to HDL-C ratio; model 2 was additionally adjusted for coffee (ml/day), total energy intake (kcal/d)

^c^Model 1 was adjusted for sex, age, BMI, TGs to HDL-C ratio, and smoking; model 2 was additionally adjusted for coffee (ml/day), dietary fat (g/d), fiber (g/d) and total energy (kcal/d)

*P < 0.05

The associations of consumption of coffee and the risk of CVD, HTN or CKD are shown in Table 4. In the crude models, compared to non-drinkers, coffee drinkers had a significant reduced risk of CVD and HTN by 54% and 41%, respectively. After adjustment for potential confounders, there was a significant inverse association between coffee consumption status and risk of CVD, with lower risk for coffee drinkers (HR = 0.58, 95% CI = 0.36–0.93). There was no significant association between coffee drinking and both HTN and CKD in the adjusted models.

**Table 4:** The risk of cardiometabolic outcomes across tertiles of coffee intakes: Tehran Lipid and Glucose Study

Coffee	Non-drinker	Drinker	*P* value	Each cup/week
CVD		(0.11–1750 ml/day)		
Crude	1.00	0.46 (0.29–0.73)^*^	0.001	0.99 (0.91–1.09)
Model 1	1.00	0.55 (0.34–0.87)^*^	0.010	1.00 (0.93–1.08)
Model 2	1.00	0.57 (0.36–0.91)^*^	0.023	1.01 (0.93–1.09)
HTN		(0.11–1750 ml/day)		
Crude	1.00	0.56 (0.44–0.73)^*^	< 0.001	0.95 (0.88–1.02)
Model 1	1.00	0.83 (0.63–1.10)	0.125	0.98 (0.92–1.04)
Model 2	1.00	0.83 (0.63–1.10)	0.121	0.98 (0.92–1.04)
CKD		(0.11–1750 ml/day)		
Crude	1.00	1.12 (0.87–1.53)	0.367	0.98 (0.93–1.04)
Model 1	1.00	1.16 (0.99–1.51)	0.249	0.97 (0.91–1.04)
Model 2	1.00	1.17 (0.90–1.51)	0.245	0.97 (0.91–1.04)

Data are hazard ratio (95% CI); proportional hazard Cox regression and logistic regression were used. *CI* confidence interval, *CKD* chronic kidney disease, *CVD* cardiovascular disease, *HTN* hypertension

Median of coffee intake among coffee drinkers was 8.33 ml/day

^a^Model 1 was adjusted for CVD risk score; model 2 was additionally adjusted for tea (ml/day), dietary fat (g/d), fiber (g/d) and total energy (kcal/d)

^b^Model 1 was adjusted for sex, age, BMI, TGs to HDL-C ratio; model 2 was additionally adjusted for tea (ml/day), total energy intake (kcal/d)

^c^Model 1 was adjusted for sex, age, BMI, TGs to HDL-C ratio, and smoking; model 2 was additionally adjusted for tea (ml/day), dietary fat (g/d), fiber (g/d) and total energy (kcal/d)

*P < 0.05

## Discussion

In our prospective population-based study, we observed that participants with higher intakes of caffeine or tea had higher incidence of CVD, whereas coffee drinking may decrease the risk of CVD events. Each 1 cup/d increased habitual consumption of tea, as well as each 100 mg/d increased caffeine intakes were related to 4% and 14% increased risk of CVD, respectively.

An inverse association between coffee consumption and CVD outcomes was documented in previous case-control studies [15, 17–19]. In a recent meta-analysis conducted by Rodríguez-Artalejo F [38], the risk of CVD decreased by 15% in subjects who consumed 3–5 cups of coffee per day, whereas coffee drinking more than 3–5 cups/d was not associated with CVD events. The protective effects of coffee could be explained by its caffeine content or other pharmacologically-bioactive compounds, such as polyphenols [39]. Contrary to conflicting results of studies focused on the health effects of caffeine, the protective effect of polyphenol-rich foods has been documented in several epidemiological studies [40–42]. Polyphenols are known to alleviate the risk of CVD by various mechanisms, including anti-inflammatory, anti-oxidant or anti-thrombotic properties of phenolic acids, as well as endothelial improvement and platelet aggregation inhibition [43–45].

Contrary to some investigations, our study found a positive relationship between tea, a popular beverage among Iranian population, and the incidence of CVD. Tea drinking was the main source of dietary caffeine (more than 90%) in our population, so it can be concluded that the adverse effect of caffeine on the risk of CVD events must be related to the high intake of tea rather than low intake of coffee. Unfortunately, we did not collect any information on the type of tea and its preparation methods that could affect the results, but previous study has been reported that tea consumed in Iran is often black tea [46], may contain a variety of additives or artificial colors, and commonly consume accompanied by sweets or sugar, while drinking green tea or coffee in many other populations is higher than black tea. The type of tea produced from the leaves depends on how the leaves are processed. For instance, fermented leaves produced black tea while non-fermented leaves create green tea. Oxidation in the process of black tea production could result in conversion flavonoids such as catechin found in green tea, into more complex varieties, which could account for different effects of black and green tea. Green tea is also thought to have a high content of vitamins and minerals [47]. The effect of black tea on blood pressure or CVD risk have investigated in a number of studies, they have shown conflicting results. Some studies have reported the protective effects of black tea, while some studies have failed to show any relationship between the intake of tea and CVD risk [48, 49]. A large prospective study in Japan reported that consumption of coffee, green tea and oolong tea, but not black tea was associated with a reduced risk of mortality from CVD [17].

Other potential mechanisms explained the difference between this studies results on the association between tea and CVD and other studies include increasing weight, impaired insulin sensitivity, increasing oxidative stress [47], due to the high amount of sweets or sugar consumed with black tea, as a common dietary habit in Iranian population. Also, demographics of subjects with highest tea intake (median intake of 1000 ml or 4 cups of tea per day) were statistically different to coffee drinkers, which could explain the different effects of tea and coffee in our population.

It is also should be noted that the risk of developing CVD or HTN in response to dietary caffeine could be related to the genetic variants of the liver enzyme Cytochrome P450 1A2 (CYP1A2), as the strongest predictors of caffeine metabolism, which may modify the association of dietary caffeine sources and incidence of CVD [50]. Also, other genetic polymorphisms in enzymes involved in metabolism or excretion of tea and coffee compounds may explain the different biological effects of them in different populations. Examples of these polymorphisms include; COMT, APO-E, GST, NADH dehydrogenase subunit 2 and methylenetetrahydrofolate reductase (MTHFR) [51, 52]. In the current study it was not possible to assess genome-wide association to examine potential genetic variants in relation to caffeine or tea and CVD. Therefore, we could not perform a Mendelian randomization study to provide an unbiased ‘causal’ estimate of the effect(s) of caffeine.

The strengths of the present study include its relatively large sample size, prospective design and long-term follow-up. In addition our study provided detailed data on potential confounders, and assessment of dietary intakes were conducted by a validated comprehensive FFQ. Use of validated CVD risk score, as the main predictor of CVD events in our population, allowed us to account for major CVD confounders without adding many variables that would lead to instability of our models. The multiple health examinations with laboratory and demographic information, allowed us to simultaneously assess three aspects of cardio-metabolic risk factors, including CVD, HTN and CKD in our population study.

Nevertheless, our study has some limitations. First, due to different dietary patterns and food habits, especially different habitual intakes of tea and coffee, we could not generalize our findings to other populations. Second, we did not have information about the type of coffee and tea, preparation methods and whether sugar or cream were added, as well as energy drinks consumption; this may have affected the results. Potential under- or overestimation of tea, coffee and caffeine intakes, due to an inherent limitation of the FFQ, can also be considered as a limitation of this study, because of which there was a potential bias in calculation and categorization of the exposure in our analysis. Although the validity of the FFQ used in the current study has been previously evaluated for energy and macro-nutrients, it was not validated for coffee, tea and caffeine. As inherent in any prospective study, some degree of misclassification might have occurred due to potential changes in the individual’s diet, as well as changes in potential confounders during the study follow-up. Finally, as in all observational studies, we could not show any causality from the results of the study.

## Conclusions

Overall, our findings support previous results regarding the protective effects of coffee intake against development of CVD; however, the results regarding the association between tea or caffeine intakes with CVD risk, investigated in our study, was contrary to those of number of previous observational studies. Further prospective studies with incorporation of different population with different dietary habits and genetic backgrounds are needed. Clinical trials also, with longer duration, are needed to confirm the associations and dose-response effects of tea, coffee and caffeine intakes with CVD, HTN and CKD.

## Abbreviations

BMI: Body Mass Index
CI: Confidence Interval
CKD: Chronic Kidney Disease
CVD: Cardio-Vascular Disease
DBP: Diastolic Blood Pressure
FFQ: Food Frequency Questionnaire
FPG: Fasting Plasma Glucose
HDL: High Density Lipoprotein
HTN: Hypertension
SBP: Systolic Blood Pressure
TG: Triglyceride
TLGS: Tehran Lipid and Glucose Study
